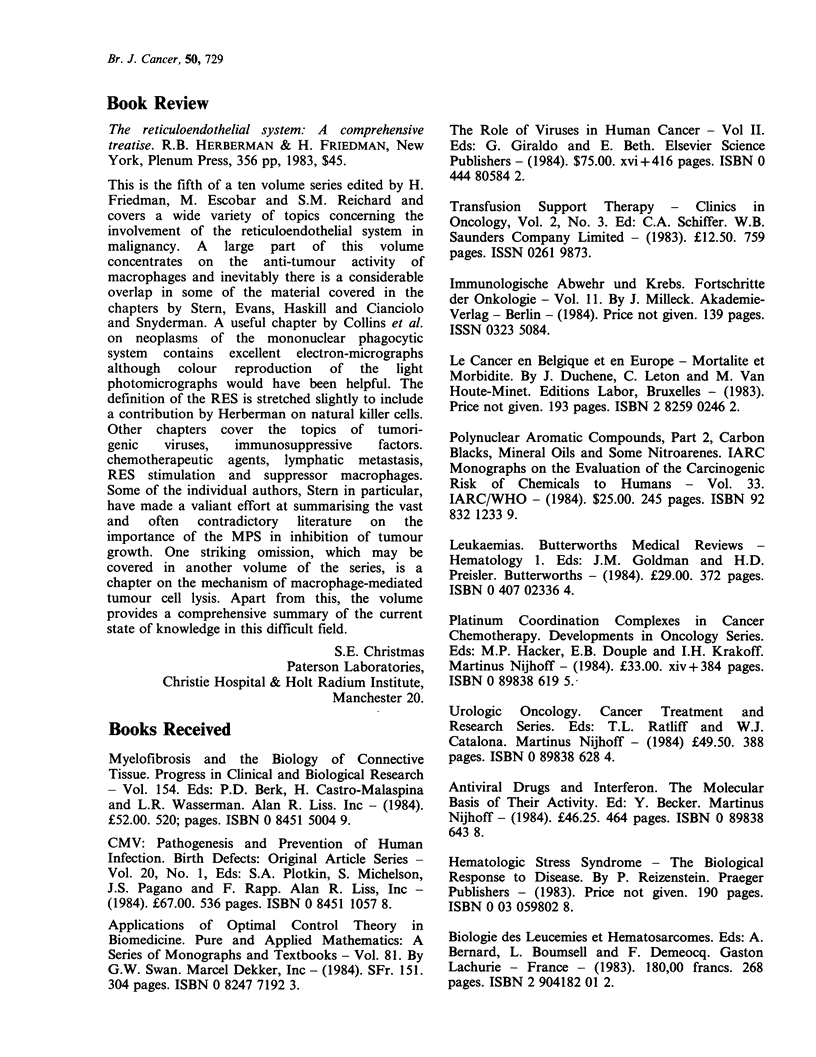# The reticuloendothelial system: A comprehensive treatise

**Published:** 1984-11

**Authors:** S.E. Christmas


### Book Review

*The reticuloendothelial system: A comprehensive treatise*. R.B. HERBERMAN & H. FRIEDMAN, New York, Plenum Press, 356 pp, 1983, $45.

This is the fifth of a ten volume series edited by H. Friedman, M. Escobar and S.M. Reichard and covers a wide variety of topics concerning the involvement of the reticuloendothelial system in malignancy. A large part of this volume concentrates on the anti-tumour activity of macrophages and inevitably there is a considerable overlap in some of the material covered in the chapters by Stern, Evans, Haskill and Cianciolo and Snyderman. A useful chapter by Collins *et al.* on neoplasms of the mononuclear phagocytic system contains excellent electron-micrographs although colour reproduction of the light photomicrographs would have been helpful. The definition of the RES is stretched slightly to include a contribution by Herberman on natural killer cells. Other chapters cover the topics of tumorigenic viruses, immunosuppressive factors. chemotherapeutic agents, lymphatic metastasis, RES stimulation and suppressor macrophages. Some of the individual authors, Stern in particular, have made a valiant effort at summarising the vast and often contradictory literature on the importance of the MPS in inhibition of tumour growth. One striking omission, which may be covered in another volume of the series, is a chapter on the mechanism of macrophage-mediated tumour cell lysis. Apart from this, the volume provides a comprehensive summary of the current state of knowledge in this difficult field.

S.E. Christmas
Paterson Laboratories,
Christie Hospital & Holt Radium Institute,
Manchester 20.